# Editorial: Mechanisms of Neuronal Migration during Corticogenesis

**DOI:** 10.3389/fnins.2016.00172

**Published:** 2016-05-03

**Authors:** Chiaki Ohtaka-Maruyama, Kazunori Nakajima, Alessandra Pierani, Nobuaki Maeda

**Affiliations:** ^1^Neural Network Project, Department of Brain Development and Neural Regeneration, Tokyo Metropolitan Institute of Medical ScienceTokyo, Japan; ^2^Department of Anatomy, Keio University School of MedicineTokyo, Japan; ^3^Centre National de la Recherche Scientifique UMR 7592, Institut Jacques Monod, Université Paris Diderot, Sorbonne Paris CitéParis, France

**Keywords:** corticogenesis, neuronal migration, neurogenesis, radial glial cells, neuronal polarization, cortical evolution, subplate, neurodevelopmental diseases

The mammalian neocortex shows an extremely well-organized structure that underlies higher brain functions such as cognition, language, and memory. The neocortex consists of a six-layered structure, in which excitatory and inhibitory neurons form complex neural circuits in concert with glial cells. As a result of recent technological innovations in live imaging and *in utero* electroporation, the processes involved in neocortical development, especially the mechanism of neuronal migration, have been successively revealed. Furthermore, it has been recognized recently that defects in neuronal migration lead to brain malformations and diverse psychiatric and neurological disorders including schizophrenia, epilepsy, and autism. Accordingly, it is important to elucidate the molecular mechanism of neuronal migration in the neocortex, in order to understand not only the basic principles of brain development but also the pathological processes of these disorders. In this special issue, we attempt to cover topics ranging from the basic mechanisms of neocortical development to the malformation and evolution of the neocortex, with a special focus on neuronal migration.

Radial glial cells (RGCs) are primary progenitors capable of generating various types of neurons and glial cells, which include Cajal-Retzius cells, subplate neurons, pyramidal neurons, interneurons, oligodendrocytes, and astrocytes. Thus, it is important to know how these diverse types of cells are generated from RGCs and integrated into complex neocortical circuits. Toma and Hanashima reviewed the mechanisms that regulate the changes in RGC competency and neuronal subtype transitions, focusing on the regulatory networks of various transcription factors including Foxg1. At the earlier stage of neocortical development, RGCs predominantly produce a large number of neurons, but later they change into glia-restricted progenitors. After the discovery of the importance of astrocytes in synaptic plasticity and blood flow, the mechanisms of glial development have attracted increasing interest for many neuroscientists. Tabata reviewed the mechanism controlling the production of diverse types of astrocytes and their migration behavior, demonstrating the multiple origins of glial cells in the neocortex.

Neocortical circuits consist of highly interconnected excitatory glutamatergic and inhibitory GABAergic neurons, which are generated from distinct pools of RGCs. The excitatory neurons are generated from RGCs localized in the ventricular zone of the dorsal telencephalon and migrate radially toward the pial surface in an inside-out manner (radial migration). On the other hand, inhibitory neurons mainly originate from the ventral telencephalon and migrate tangentially into the neocortex (tangential migration). In spite of such different developmental origins, both excitatory and inhibitory neurons go through the multipolar stage with several minor processes in the neocortex before axon extension. Then, they undergo dramatic morphological changes to initiate axon formation, namely, neuronal polarization. Sakakibara and Hatanaka reviewed the sequential events in polarization processes of both excitatory and inhibitory neurons, and they discussed the underlying molecular mechanisms.

At the multipolar stage, the excitatory neurons transiently use a multipolar migration mode, namely migration with no fixed direction, in the subventricular and intermediate zones. Then, they adopt a bipolar shape during neuronal polarization and migrate quickly toward the pial surface along RGC processes, which is called locomotion mode. Many kinds of molecules are involved in these dynamic changes in the morphology and behavior of neurons. Small GTP binding proteins belonging to the Rho family play critical roles in cytoskeletal regulation during such dynamic processes. Azzarelli et al. reviewed the roles of Rnd proteins, “atypical” Rho family members, in neuronal migration and discussed its upstream and downstream pathways. The functions of many cytoplasmic proteins including cytoskeletal components are regulated by phosphorylation and dephosphorylation processes. Ohshima focused on protein kinases, including CDK5 and JNKs, and reviewed their regulatory roles in cytoskeletal organization during multipolar-bipolar transition and radial migration. Ohtaka-Maruyama and Okado comprehensively summarized the molecular pathways involved in these developmental processes, emphasizing the importance of subplate neurons in the development and evolution of the six-layered neocortical structure.

It is apparent that neuronal migration and wiring are regulated by various secreted factors such as growth factors, chemokines, and extracellular matrix molecules, although their mechanisms are poorly understood. Kondo et al. demonstrated that subplate neurons transiently express high levels of secretary proteins such as connective tissue growth factor, neuroserpin, and insulin-like growth factor binding protein 5, which may be involved in cortical circuit formation. Greenman et al. reported a novel finding that autotaxin (ENPP2), a secretary enzyme bearing lysophospholipase D activity, regulates the localization and adhesion of neural progenitor cells independent of its catalytic activity. Maeda reviewed the roles of proteoglycans in neuronal polarization and migration and discussed the possibility that extracellular matrix regulates the distribution and activity of multiple secreted factors in the developing neocortex. In addition to the long-range gradient of secreted factors, axon pathfinding is also regulated by short-range guidance cues and direct cell-cell contacts mediated by guidepost cells. Squarzoni et al. reviewed the roles of already known guideposts such as Cajal-Retzius cells for entorhinal-hippocampal axons and corridor cells for thalamocortical axons, and further proposed a new class of guidepost cells, microglia, in the cortex.

Hippocampal formation has a close relationship with the neocortex both functionally and structurally, but it shows a distinct arrangement of pyramidal neurons from that of the neocortex. Hayashi et al. reviewed the differences in the migratory behaviors of neocortical and hippocampal neurons, which lead to the formation of distinct layered structures in these two cortical regions. Defects in the migration of excitatory and inhibitory neurons can lead to the various neurological and psychiatric disorders. Kato reviewed recent development in the understanding of the genetic bases of neuronal migration disorders in terms of genotype-phenotype correlations, focusing mainly on lissencepahaly. Muraki and Tanigaki discussed the possible relationship between neuronal migration defects and behavioral abnormalities relevant to schizophrenia based on studies using genetically defined animal models. The evolutionary approaches should greatly deepen our understanding of the mechanisms underlying neocortical development. Nomura et al. established the method of *in ovo* electroporation and *ex ovo* culture of reptilian embryos. Comparative studies using this method will provide significant insights into the origin of the mammalian neocortex.

It is hoped that the special issue entitled “Mechanisms of Neuronal Migration during Corticogenesis” will serve as a valuable resource for many neuroscientists to promote their research perspectives. Finally, as topic editors, we would like to express our sincere appreciation to all the authors for their outstanding contributions and to all the reviewers for their insightful comments on the papers. We also thank the editorial office and the production staff for their unceasing efforts and dedication.

## Author contributions

CO wrote the manuscript. KN, AP, and NM revised the manuscript.

### Conflict of interest statement

The authors declare that the research was conducted in the absence of any commercial or financial relationships that could be construed as a potential conflict of interest.

